# On the Origin and Characteristics of Noise-Induced Lévy Walks of *E. Coli*

**DOI:** 10.1371/journal.pone.0018623

**Published:** 2011-04-08

**Authors:** Franziska Matthäus, Mario S. Mommer, Tine Curk, Jure Dobnikar

**Affiliations:** 1 Center for Modeling and Simulation in the Biosciences (BIOMS), University of Heidelberg, Heidelberg, Germany; 2 Interdisciplinary Center for Scientific Computing (IWR), University of Heidelberg, Heidelberg, Germany; 3 Faculty of Natural Sciences and Mathematics, University of Maribor, Maribor, Slovenia; 4 Department of Chemistry, University of Cambridge, Cambridge, United Kingdom; 5 Department of Theoretical Physics, Jožef Stefan Institute, Ljubljana, Slovenia; Memorial Sloan Kettering Cancer Center, United States of America

## Abstract

Lévy walks as a random search strategy have recently attracted a lot of attention, and have been described in many animal species. However, very little is known about one of the most important issues, namely how Lévy walks are generated by biological organisms. We study a model of the chemotaxis signaling pathway of *E. coli*, and demonstrate that stochastic fluctuations and the specific design of the signaling pathway in concert enable the generation of Lévy walks. We show that Lévy walks result from the superposition of an ensemble of exponential distributions, which occurs due to the shifts in the internal enzyme concentrations following the stochastic fluctuations. With our approach we derive the power-law analytically from a model of the chemotaxis signaling pathway, and obtain a power-law exponent 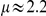, which coincides with experimental results. This work provides a means to confirm Lévy walks as natural phenomenon by providing understanding on the process through which they emerge. Furthermore, our results give novel insights into the design aspects of biological systems that are capable of translating additive noise on the microscopic scale into beneficial macroscopic behavior.

## Introduction

The motion of *E. coli* bacteria is characterized by a sequence of run and tumble events [1]. During a run the flagella of the bacteria rotate counter-clockwise, form a bundle and propel the cell in a more or less straight line. If the flagella rotate clockwise, the bundle opens and the bacteria randomly change their angle of motion without forward propagation (tumble). The switching between these two events (clockwise and counter-clockwise rotation) is controlled by the chemotaxis signaling pathway. Solely by adjusting their switching rate are *E. coli* bacteria capable of responding to their environment. For instance, through a very long run not interrupted by a change of direction the bacteria can swim away from a hostile environment. In a neutral environment without nutrients the bacteria are generally believed to switch between running and tumbling in such a way as to perform diffusive search for food. Recently, however, there has been experimental and theoretical indication [2], [3] that stochastic fluctuations affecting the signaling processes can enable the bacteria to perform Lévy walks, a motion which corresponds to superdiffusion on the macro-scale. In an earlier paper [3] we have shown that there is an evolutionary advantage of performing Lévy walks in food deprived environments. In this article we will now address a very important related question, namely, how noisy fluctuations can actually lead to a transition from classical random walk to Lévy walks. This phenomenon has a fascinating broader implication, as it represents a case where additive noise on the concentration level induces a change in the macroscopic behavior of the organism, which gives evolutionary advantage under some conditions. The bacterium *E. coli* shows a broad variety of behaviors that are governed by rather simple and well understood signaling pathways, which makes it an ideal model organism for experimental and theoretical research. For the same reason *E. coli* seems to be the optimal choice for our theoretical investigation aimed at understanding the internal mechanisms of a biological organism that is capable of generating superdiffusive behavior.

We describe the motion of *E. coli* in a coarse-grained way, as composed of consecutive run and tumble events with the tumbling probability derived at each step from a model of the chemotactic signaling pathway (see Methods or [3]). If the concentration dynamics of CheR (the enzyme regulating the receptor methylation) is described as a stochastic process, the model predicts a power-law run length distribution. We show that the power-laws result from nonlinear properties of the signaling pathway coupled with a variable level of the chemotactic enzyme CheR. The CheR variations propagate through the signaling cascade and effectively lead to a superposition of an ensemble of exponential run length distributions in a way that a power-law run length distribution results. Based on steady state relations between the system variables we analytically relate the properties of the CheR variation to the form of the run length distribution. We discuss the conditions under which power-laws are expected and obtain, under very moderate additional assumptions, the exponent 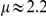, which corresponds to the experimentally determined exponent from [2]. Further numerical simulations strongly support the analytical result.

Our approach significantly extends previous work by Tu and Grinstein [4], who showed that temporal fluctuations in CheYp, the enzyme directly controlling the flagellar motors, lead to power-law run length distributions. Our approach generalizes this finding as we consider the entire chemotaxis signaling pathway and describe the origin of the fluctuations. We also investigate the effects of the rotational diffusion on the run length statistics.

### Lévy walks in nature

A very intense ongoing discussion in the scientific literature concerns the question whether some animal species use Lévy walks as a random search strategy to localize prey, food or mating partners. A Lévy walk is a scale-invariant type of motion characterized by a power-law run length distribution 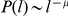 with 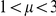. Lévy walk like behavior has been reported in a number of species, ranging from zooplankton [5] via marine predators [6] and spider monkeys [7], to humans [8]. However, in some cases refined measurements of animal movement did not support previously collected data. In the case of the albatross, for instance, a device attached to their legs measured the lengths of wet and dry periods - wet related to feeding, dry to flying [9]. The apparent power-law, which was initially reported, disappeared upon including the information that some of the long flights were actually periods when the birds were still located on the island [10]. In other cases Lévy walks were detected by plotting the histogram on double-logarithmic axis, which is not a very reliable way to determine the power-law exponents [11]. Estimating the exponents with more reliable methods revealed exponents with 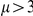, and thus a finite variance in the degree distribution (see, for instance, [10]). These findings heated up the debate on Lévy walks in animal species, and up to now there has been no consensus or general agreement on whether or not this type of motion is common in biological systems.

### How optimal are Lévy walks?

Another vividly discussed topic is to what extent a Lévy walk presents an optimal search strategy. This is an important question, because if Lévy walks do present an evolutionary advantage in some situations, then it is likely for at least some species to have developed this type of search strategy. When addressing the issue of optimality the difficulty lies in the definition of the term optimal, and in the question what other search strategy the Lévy walk is compared to. Bartumeus [12] finds that a Lévy walk is more efficient than a Brownian walk when optimality is measured as the number of animal interactions. Benhamou [13], however, argues that in a patchy habitat a composite Brownian search strategy (long runs between patches and short runs within) outperforms a Lévy walk. This strategy, however, uses additional information: the random walker **knows** whether he is inside or outside a patch, and actively changes his strategy according to the location. Obviously, the more information is available and the better the information can be processed, the more efficient is the search strategy. An “intelligent” random walker, which can choose between a variety of behaviors according to the situation, or is capable of sensing the target (for instance through chemotaxis [14], vision or smell), will certainly outperform a random walker lacking these capabilities.

### Evidence for Lévy walks in *E. coli*

In the seminal paper of Berg et al. [1] the run length distribution of *E. coli* is accessed through bacterial tracking. The authors conclude that the run length distribution is exponential, however, they also comment that the histogram of the run lengths does not give a perfectly straight line on a semi-logarithmic plot. In an article of Wu et al. [15] a method for three-dimensional tracking of *E. coli* bacteria is presented. An analysis of the thus accessed trajectory revealed that superdiffusive motion is present for a certain time scale (up to 3.6 seconds), after which a transition to classical diffusion occurs. The best indication for power-laws in the *E. coli* run length distribution was given by Korobkova et al. [2]. Here, the bacteria are not tracked, but the time intervals of clockwise and counter-clockwise rotation of flagella are recorded, so they were able to obtain very long time series for every bacterium and could therefore reveal rotation times that lasted over 15 seconds. The distribution of the counter clockwise (CCW) intervals was power-law, but truncated for very long run lengths. The power-law in the run length distribution disappeared when CheR was over-expressed, indicating that fluctuations affecting CheR alone control the functional form of the run length distribution.

The experimental findings in [2] were supported by stochastic simulations of the chemotaxis signaling pathway using StochSim [16]. Through these molecular-level simulations the power-law form of the CCW events could be reproduced for low CheR levels and disappeared for increased concentrations. On a more coarse level, the chemotactic signaling pathway of *E. coli* can be described by a system of coupled ordinary differential and algebraic equations [3], [17]. Numerical simulations based on this model [3] also confirmed the emergence of power-law run length distributions when CheR, an enzyme regulating receptor methylation, is affected by stochastic fluctuations.

## Results

We adapted and implemented a model of the chemotaxis signaling pathway [3], where the levels of the chemotactic proteins and the response of the molecular motors responsible for the rotation of the flagella are described by a system of differential and algebraic equations (for details we refer to the Methods section). The output of the model is the time dependent probability of tumbling 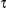. We evaluated 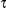 for each bacterium at discrete simulation times and made the bacteria run or tumble in the next step according to these probabilities, defining a modulated Bernoulli process with a corresponding constant time interval 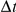 between draws. The simulation output therefore consists of a sequence of swimming and tumbling events, each with a fixed duration 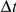. The speed of swimming 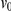 is assumed to be constant, therefore each swimming step has a length 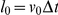. The bacterial trajectories between two tumbles are straight lines (the effects of rotational diffusion are discussed in a later section), and we can measure the length of each such segment: 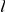 consecutive runs, not interrupted by a tumble, are seen as a single (coarse) run of length 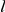. In this article we focus on the distribution of these run lengths, and investigate the influence of the system parameters and CheR-related stochastic fluctuations on its functional form.

The stochasticity in the signaling pathway finds its origin in binding and dissociation process of CheR to and from the receptor [3]. Of all the enzymes involved in the chemotaxis signaling pathway, CheR exhibits the lowest concentration, and it has been shown that over-expression of CheR makes the power-law disappear. Fluctuations in binding and dissociation result in a temporally varying amount of CheR being bound to the receptor, and thus induce temporal changes in the methylation level. In modeling, such variation is easiest achieved by assuming that the overall CheR concentration varies and therefore we have added a fluctuating term to the CheR level in our model:
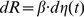
(1)with 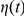 representing a Wiener process and 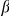 is the corresponding noise parameter. This equation describes Brownian noise, characterized by low frequency modes. Brownian noise is non-stationary, so 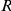 must be bounded from below to guarantee non-negativity, and for symmetry reasons also from above. Thus, the CheR concentration performs a random walk within a bounded interval. Instead of the Wiener process given by the Equation (1), a slightly different random process was used in [3]. In order to compare the numerical values of the parameter 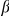 in both models, it needs to be rescaled: 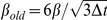.

For a constant CheR concentration, with the signaling pathway at steady state, and in the absence of chemoattractants, the model yields an exponential run length distribution corresponding to classical diffusion. From [3] we empirically know that at steady state the average jump length 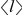 depends on the (fixed) CheR concentration 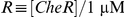 as
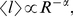
(2)with 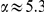 at the biologically relevant values of the pathway parameters [3].

The chemotaxis signaling pathway model extended by Equation (1) yields a power-law run length distribution [3]. Further investigations showed that the power-law run length distribution shows robustness with respect to the parameter 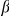, and that it persists over several magnitudes of run lengths, also when instead of the run length distribution the cumulative (survival probability, histogram of steps of length 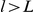) is plotted. Furthermore, the power-law is also robust with respect to the noise model. To demonstrate this we additionally modeled the noise dynamics by an Ornstein-Uhlenbeck process, given as
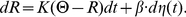
(3)Like in the case of the confined Brownian noise (Equation 1), we observed power-laws for a wide range of parameters 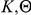 and 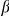.

### Steady state model of run and tumble motion

For a constant CheR concentration, the system of equations describing the signal transduction is deterministic and, hence, the resulting tumbling probability 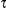 is constant. The probability of making a jump of length 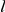 is then 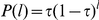, since a jump of length 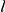 consists of 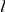 consecutive swims and a final tumble. For small 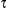 the expression can be approximated by
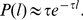
(4)


After many jumps the average run length is given as 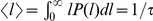. Combining the empirical result of numerical simulations (2) and the relation 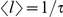 we get
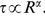
(5)


At each fixed value of CheR we thus predict an exponential run length distribution (4), but the tumbling probability depends on the CheR level in a nonlinear way as specified in (5).

### Superposing exponentials

**Figure 1:**
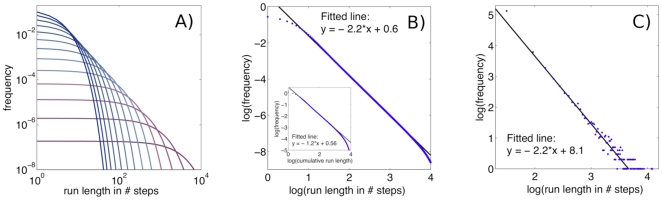
Power-law run length distributions obtained by superposing exponential distributions. **A**): Nine exponential run length distributions for random walks with constant tumbling probabilities 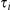. The curves are plotted on double-logarithmic axes to demonstrate the power-law that results from their superposition. **B**): The superposition of the nine exponential distributions from A) leading to a power-law distribution (blue dots) with the exponent 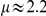 obtained by fitting (solid black line). The cumulative distribution of the same data is plotted in the inset. **C**): Combined run length distribution (blue dots) of the 9 bacteria from numerical simulations with constant CheR levels (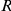 between 0.04 and 0.12, corresponding to the 9 values of 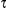 from A)). The fitted exponent (solid black line) is again 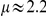.

Now we consider the combined distribution of run lengths of several diffusive processes with constant but different values of 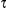. It is known that such a superposition can in principle result in a power-law distribution [18], a fact that has already been used to model subdiffusive transport [19]. We have performed a simple supporting test: we determined the exponential run length distributions for nine different values of 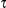 (Figure 1A) and superposed them. We obtained a power-law distribution spanning many orders of magnitude (Figure 1B) with an exponent of approximately 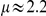. Naturally, a superposition of a limited number of exponentials can result in a power-law distribution only in a certain range, after which there is a truncation (here the range is 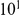 to 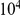). Additionally, we performed numerical simulations with nine bacteria having constant CheR levels corresponding exactly to the nine values of 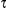. Figure 1C shows their combined run length distribution showing good agreement with the simple model prediction in Figure 1B.

### Derivation of the power-law

Let us now consider a situation in which the concentration of CheR in a single cell changes very slowly with time around its average value. The system goes through consecutive states, each featuring an exponential distribution with a different average run length. Thus, the run length distribution averaged over a certain time interval is again a sum of different exponentials, each weighted by its probability of being chosen. Therefore, if the distribution of the CheR values and the model parameters are appropriate, we can expect to observe power-law run length distributions.

If the CheR variation 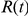 is slow enough, the tumbling probability is constant over time intervals 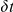 much longer than the time step 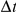 and the cell can make several coarse runs with almost constant 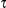. The number of separate runs 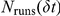 (with variable lengths 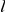) within this interval is the ratio between the sum of all run lengths 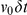 and the average run length 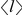 at the given 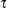. The number of runs per unit of time is then
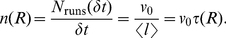
(6)


The distribution of the run lengths 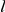 observed after a long time 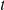 is then defined as
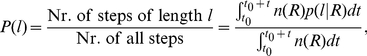
(7)where 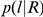 is the conditional probability (Equation 4) for making a run of length 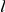 at a given time (with a given 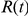). Instead of over 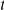 we want to integrate over 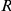, therefore we replace 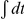 with 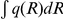, where 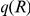 measures the distribution of the 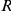 values during the whole period 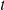. We then have:
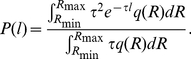
(8)


In the special case when 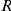 is uniformly distributed in an interval, it follows that
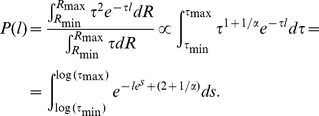
(9)


The integral on the right corresponds to a truncated version of an expression for 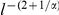,
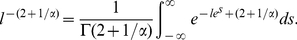
(10)


Truncated versions of this integral were used in [18] to produce accurate approximations of power-laws in arbitrarily long intervals (through the use of a quadrature rule to evaluate (9) with 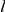 left as a free variable, we obtain an approximation to 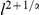 as a sum of exponentials). If we ignore that 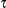 is a probability, and thus its values are constrained to the interval 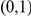, we can extend the integration limits. When evaluating the integrals 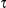 is multiplied by 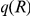 which is nonzero only for values of 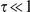. The wild type average CheR concentration is about 0.16 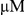, therefore the values of 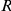 (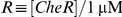) in our model are typically 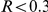. Given the relation (Equation 5) this translates to values of 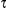 bellow 0.01. Therefore, even if we formally extend the limits of integration to 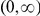, we do not alter the result. The Equation (9) then becomes
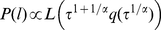
(11)with 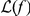 being the Laplace transform of the function 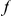. By evaluating the above expression we can compare 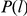 resulting from various 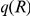 and thus relate the properties of the CheR signal to the distribution of the run lengths. Table 1 contains analytical results obtained from (11) for a few representative distributions of CheR values. We see that, as long as the distribution of the CheR levels 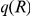 is close enough to uniform, we expect the power-law scaling of 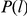 with the exponent
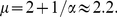
(12)


**Table 1:** Run length distribution corresponding to different distributions of the CheR concentration level.

Distribution of CheR levels 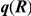	Distribution of run lengths 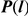	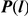 for 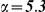
Constant CheR: 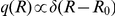	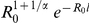	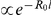
Uniform Distribution: 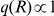	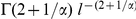	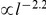
Gaussian Distribution: 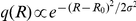	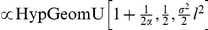	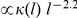 ; 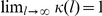

Our approach here is similar to that of Tu and Grinstein [4]. One important difference, however, is that we start directly from fluctuations affecting CheR which have been shown to control the power-law. We thus involve in our analysis the entire chemotaxis signaling pathway, and therefore achieve a better estimate of the power-law exponent, coinciding with experimental measurements [2]. Furthermore, we leave the exact shape of the probability density function of CheR unspecified until a later step, which allows us to consider different fluctuation patterns and to obtain additional information on the robustness of the phenomenon.

### Limits of the steady state result

**Figure 2:**
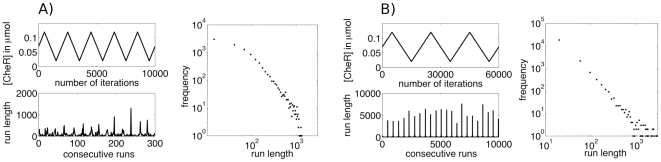
Run length distributions from a numerical simulation of *E. coli* in nutrient free environment with deterministic and periodic (zigzag) CheR dynamics with uniform distribution 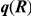 on the interval 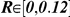. Both figures contain three panels. The upper left shows the CheR level variation 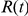, the lower left the correlation between consecutive run lengths 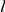 and the right one the distribution of the run lengths 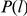. Signals with two different frequencies are compared: **A**) relatively high frequency and **B**) relatively low frequency. The power-law run length distributions extends over at least two decades in B) while it is confined to about one decade in A).

Now, for a single bacterium with stochastically fluctuating CheR, one can expect Equation (11) and Table 1 to hold. Thus, we expect a power-law run length distribution if the distribution of the values of 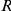 is close to uniform and if the variations are slow enough to render the steady state assumption plausible. If the CheR variation is too fast, the steady state assumption is violated and we expect deviations. To demonstrate the influence of the low frequency modes on the power-law distribution we have conducted a very simple simulation with “deterministic fluctuations” of CheR. In Figure 2 we show the run length distribution for simulated bacteria with CheR following a zigzag curve, which has a uniform distribution of 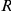 values in a given interval. We compare two CheR signals: one with relatively long period (Figure 2B) and one with about 10 times shorter period (Figure 2A). We see the power-law behavior in both cases, however for the faster oscillating CheR signal the truncation is much more significant. The range over which the power-law extends is also reduced if the amplitude of the zigzag is smaller. This simulation shows that a power-law run length distribution can result from purely deterministic dynamics of CheR, the stochasticity not being a necessary condition.

### Effects of rotational diffusion

**Figure 3:**
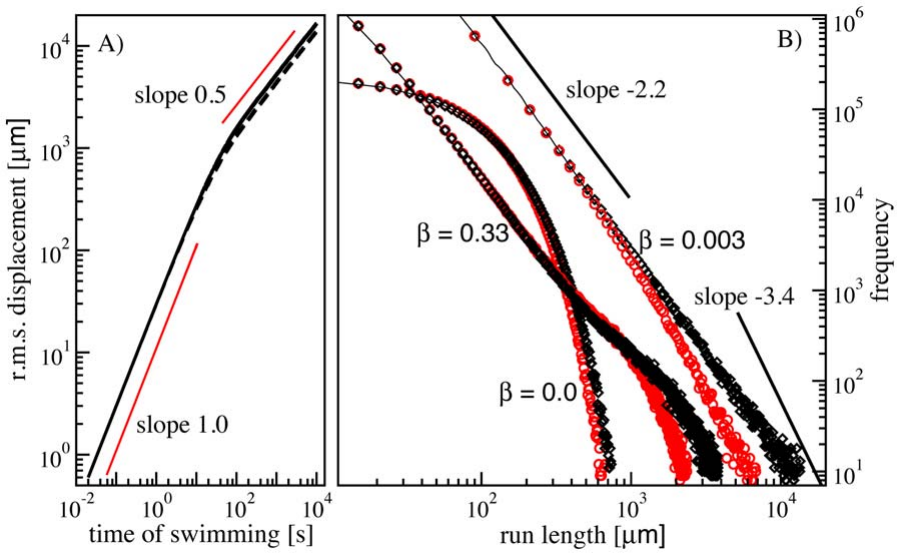
Effect of rotational diffusion on the run length statistics of *E. coli*. **A**) Dependence of the effective run length (root mean square displacement) on the time of swimming for a single trajectory with rotational diffusion. The two curves are in 2D (solid curve) and in 3D (dashed curve). In 3D the diffusive regime sets in slightly earlier because the rotational diffusion works on two angles and is consequently more pronounced. However, the crossover regime is in both cases around 20 s or 30 s, which corresponds to swims of about 1 mm. **B**) Run length distributions from numerical simulations of *E. coli* in nutrient free environment. The confined Brownian CheR dynamics is given by Eq. (1) with CheR fluctuating around an average of 0.07 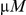 (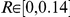). Three different values of the parameter 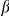 are compared: 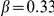, 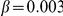, and 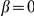. For each value of 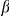 we show simulation results with (red circles) and without (black diamonds) rotational diffusion. The run length distribution is exponential when no noise is added (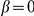) and power-law with the exponent about 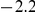 at nonzero 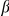. For the smaller value 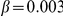 where slow modes in noise fluctuations are prominent, the power-law persists over a larger range, while at large 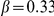 the distribution features an exponential cut-off. The effect of the rotational diffusion is an earlier cut-off after which the power-law exponent changes to about 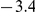.

Bacteria do not change the direction of motion only due to tumbling, but also due to collisions with the surrounding medium, which are governed by thermal fluctuations. Such collisions exert random torque on the cells and cause their trajectories to deviate from straight lines. This phenomena, called rotational diffusion, was quantified by Berg et al. in [1], who measured the angular diffusion constant 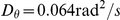. Rotational diffusion causes the straight trajectories to bend and the correlation between the initial and final swimming direction is lost after a typical time 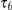, which is between 10 s and 100 s (see Figure 3A). For times shorter than 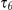 the trajectories appear ballistic, while for 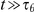 they are diffusive. The actual run length 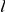 for a swim of length 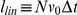 is therefore equal to 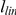 for 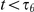 and it becomes proportional to 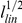 for long trajectories. This effective reduction of the run lengths on large scales has an impact on the predicted run length distributions 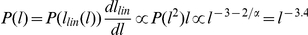. A distribution with a power-law exponent of 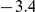 has a finite second moment and does not describe a super-diffusive process. The rotational diffusion therefore sets a constraint on the length scales over which we can expect to observe super-diffusive behavior. From Figure 3A we can conclude that this length scale corresponds to swims of about 20 s to 30 s, (run lengths of about 0.6 mm to 1 mm). We have additionally confirmed this (Figure 3B) by evaluating the run length distributions in numerical simulations of bacteria with and without rotational diffusion. One can see that rotational diffusion introduces a cut-off, but the observed power laws nevertheless persist over several orders of magnitude.

## Discussion

### Slow modes

We observe power-law run length distributions predicted by (11) and Table 1 when the fluctuations exhibit low-frequency modes causing slow changes in the CheR level and the concentration of CheR is sampled almost uniformly in a certain interval. The low-frequency modes of the noise guarantee slow fluctuations and are essential because the fluctuations in CheR must be translated into fluctuations in phosphorylated CheY, the control enzyme for the flagellar motor, to result in changes in the tumbling frequency. A change in CheR is followed by a shift in the methylation level, and thus in the probability of the receptor to be in the active/inactive state. Since the methylation process has a certain relaxation time, the chemotaxis signaling pathway functions like a low-pass filter, smoothing out high frequency modes of the CheR fluctuations. To change the tumbling frequency significantly, the internal concentration levels must have time to adapt to a change in the level of CheR, and this is only possible if the level of CheR stays above or below average for a longer time. Consequently, this enables a large variability in the run lengths and eventually results in a Lévy walk. The requirement of the low-frequency modes is met when the dynamics of CheR is modeled by bounded Brownian noise (Eq. (1)). The exponential return times, a characteristic of Brownian noise, yield long intervals in which CheR can be above or below average. Hereby, the parameter 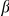 has important influence on the dynamics of the fluctuations and should neither be too large nor too small. If 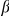 is very large, then the resulting variations in the CheR level are too fast for the internal kinetics of the chemotactic enzymes to follow. Very small 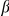, on the other hand, cause very slow changes in the dynamics of CheR, which might appear constant on the time scale of a process the bacteria are involved in, on the typical time scale of observation or even on the time scale of a bacterial life span. The results in Figure 3A confirm this as one can see power-laws in a significant range for slowly varying CheR. If, instead of Brownian noise, we model the dynamics of CheR by an Ornstein-Uhlenbeck process, then the parameters 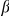, 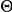 and 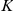 affect the noise spectrum, and therefore must be in a certain range and relation to each other. It is not possible to give fixed boundaries for the intervals from which the parameters must be chosen, because there is a smooth transition in which the range of the observed power-law gradually reduces.

### Truncation

A variety of factors ensure that the lengths of swimming distances are bounded, the most obvious one being the finite life of the bacteria. Also, the range of exponential run length distributions that can be sampled is limited by the finite number of receptors and the limited number of times each receptor can be methylated. The upper bound in run length has as the consequence that the power law distributions are truncated, decaying exponentially for long distances. As we have shown above, the characteristics of the fluctuations of CheR also determine the time scales for which power laws can be observed. If the fluctuations are too fast or their amplitude too low, or if the fluctuations sample the concentration in a very biased way, no power law will be visible at all. Finally, we have also quantified the effects of the rotational diffusion on the run length distribution and showed that there is some additional truncation due to this effect, however, the power law distribution can still be observed over several orders of magnitude.

### Lévy walks in bacterial populations?

In real populations of *E. coli* bacteria the level of CheR varies from one individual to another. Presumably, the stochastic fluctuations in the CheR level are more important in the individuals with a lower CheR expression. Recently it has been experimentally verified [20] that larger behavioral variability (fluctuations in the CheYp level) relates to stronger cellular response (chemotactic drift). Our results can be seen as an addition to this conclusion, showing first how the CheYp variations are generated by the chemotactic signaling pathway from stochastic processes involved in CheR regulation, and second that the cells with stronger response to stimuli can sometimes perform Levy walks when the stimuli are not present. Due to the cell to cell variability it is to be expected that in real *E. coli* populations only some bacteria perform Levy walks, others classical random walks, and that many bacteria may show intermediate behavior.

### Summary

We have used a model of the *E. coli* chemotaxis signaling pathway to show how the variations of the CheR level propagate through the signal transduction network. Our results suggest, that the power-law in the run length distribution origins from a superposition of exponential distributions, each related to the run length distribution for a constant concentration of CheR. Following this superposition assumption we are able to derive the exponent of the power-law analytically. The result, 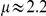, agrees well with the additionally performed simulations where the noisy CheR signal was modeled either by the confined Brownian noise or by an Ornstein-Uhlenbeck process, and fits also to the exponent obtained from experimental approaches [2]. The power-laws obtained in our simulations are truncated, since the power-law relationship between CheR and the tumbling frequency 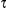 holds only in a certain range but the distribution of the run lengths is well described by a power-law over a range spanning several magnitudes. The frequency distribution of the noise is influencing this range, but not the exponent of the power-law. Prominent low frequencies cause the power-law to extend over a wider range than a fluctuations characterized by high frequencies. In an earlier paper, Benhamou [13] discussed the advantageous of a composite random walk, with either shorter or longer runs connected to the structure of the landscape. Here one can see that if this idea is taken a step further, considering a continuous ensemble of classical random walks, a Lévy walk can result if the sampling of the classical random walks follows certain criteria. In our case the sampling is determined by the CheR fluctuations, and independent on the location of the bacteria.

This work provides novel insights into noise as a **positive trait**, actively exploited by a biological system to gain evolutionary advantage. The prevailing view is rather opposite: noise represents a **negative**, but unfortunately inevitable, phenomenon, and biological organisms have evolved to show robustness to it. In the past decade, however, research in very different areas has revealed a number of mechanisms in which noise actually proves beneficial for the considered organism. One rather evident advantage is that stochastic fluctuations can introduce heterogeneity into a genetically identical population [21], and thus, for instance facilitate adaptation in a variable environment. A further, not so obvious, result is that stochastic fluctuations can be exploited to induce switching in bi- or multistable systems, a phenomenon that seems to be involved in stem cell differentiation [22], [23], or in the decision processes involved in the lysis-lysogeny cycle for phage 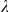-infected *E. coli cells* [24]. In [25] it has been shown that multiplicative noise, arising from fluctuations in protein degradation processes, does not only induce switching, but also enables synchronized switching within a large population of bacteria. Yet another benefit arising from stochastic fluctuations was presented in [26], where simple gene-regulatory networks were constructed for which multiplicative noise led to an amplification in the gene expression. In our case, stochastic fluctuations on the concentration level lead to a change in the macroscopic behavior, and this behavior presents a more efficient strategy than a classical random walk when searching for randomly located and sparse nutrition sources. Most fascinating in this case is that not only the presence of noise, but also, and even more, the specific interactions of the regulating enzymes are the prerequisite for the generation of Lévy walks.

## Methods

### Chemotactic signaling pathway

The chemotactic signaling pathway is a network of chemical reactions propagating signals from the receptors to the flagellar motors. The state of the network depends on its history, which effectively introduces memory into the signaling pathway and in this way bacteria can estimate food gradients. Involved in the pathway are: a membrane receptor, a methylation cascade influencing the state of the receptor, and a phosphorylation cascade, transmitting the information on the receptor state to the flagellar motors [17]. The chemotaxis receptor is believed to switch between two states [27], [28] - active an inactive, where the active state induces a tumble and the inactive state a swim. Methylation of the receptor increases its probability to be in the active state. Thus, via methylation and de-methylation processes an adaptation of the run lengths to different absolute ligand concentrations can be achieved. The two enzymes controlling the methylation cascade are called CheB (controlling de-methylation) and CheR (controlling methylation). The model we used to quantitatively describe the signaling pathway is the model 1c from Kollmann et al. [17]. The dynamics of the protein levels in the cell are described by a system of coupled ordinary nonlinear differential equations and the resulting level of the phosphorylated protein CheYp is coupled to the motor switching curve regulating the sense of flagellar rotation and through it the probability for swimming or tumbling. The model and all the parameter values are specified in detail in [3].

The starting point for the analysis conducted here is a result from our previous work [3], where we obtained the steady-state relation between the CheR level in the cell 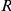 and the probability 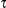 of tumbling in a nutrient-free environment. Since most of the interactions within the signaling pathway are described by Michaelis-Menten kinetics the differential equations are nonlinear and the resulting relation is a power-law:
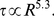
(13)


### Robustness with respect to kinetic parameters

The kinetic parameters enter our derivation of the run-length distribution through their influence on the exponent 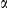, which defines the the steady-state relation between the level of CheR and the average run length distribution (2). The parameter 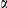 influences the exponent of the resulting power-law, as 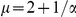. We know that a Lévy walk is characterized by a power-law run length distribution with an exponent 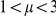. From the relationship 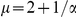 we see that the Lévy walk behavior will persist as long as 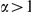. Even more, for any 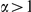, we have 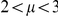, which is the interesting range for which the first moment of the power-law run length distribution converges and only the second moment diverges. The qualitative behavior of the Lévy walk is not affected, even when variations in the kinetic parameters lead to a five-fold decrease in 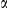. Parameter variations that lead to an increase of 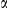, irrespective of how strong the increase, can not drive the bacterial motion behavior from being a Lévy walk.
